# The genus *Macroteleia* Westwood in Middle Miocene amber from Peru (Hymenoptera, Platygastridae *s.l.*, Scelioninae)

**DOI:** 10.3897/zookeys.426.7822

**Published:** 2014-06-17

**Authors:** Vincent Perrichot, Pierre-Olivier Antoine, Rodolfo Salas-Gismondi, John J. Flynn, Michael S. Engel

**Affiliations:** 1 CNRS UMR 6118 Géosciences and Observatoire des Sciences de l’Univers de Rennes, Université Rennes 1, Campus de Beaulieu bât. 15, 263 avenue du Général Leclerc, 35042 Rennes Cedex, France; 2 University of Kansas Biodiversity Institute, Lawrence, Kansas 66045, USA; 3 Institut des Sciences de l’Évolution, Université Montpellier 2-CNRS-IRD, F-34095 Montpellier, France; 4 Departamento de Paleontología de Vertebrados, Museo de Historia Natural, Universidad Nacional Mayor de San Marcos, Lima, Peru; 5 Division of Paleontology, American Museum of Natural History, Central Park West at 79th Street, New York, NY 10024-5192, USA; 6 Division of Entomology (Paleoentomology), Natural History Museum, and Department of Ecology & Evolutionary Biology, 1501 Crestline Drive – Suite 140, University of Kansas, Lawrence, Kansas 66045, USA

**Keywords:** Insecta, Platygastroidea, *Macroteleia*, Tertiary, Neogene, Peru, Amazonian amber, taxonomy

## Abstract

A new species of the scelionine genus *Macroteleia* Westwood (Platygastridae *s.l.*, Scelioninae) is described and figured from a female beautifully preserved in Middle Miocene amber from Peru. *Macroteleia yaguarum* Perrichot & Engel, **sp. n.**, shows a unique combination of characters otherwise seen independently within its congeners. It is most similar to the modern *M. surfacei* Brues, but differs from it by the non-foveolate notauli, the contiguous punctures of the vertex, and the continuous propodeum. The new species is the first New World fossil of the genus, suggesting a Cretaceous origin for the group and a relatively old age of the South American, tropical African, and Australian faunas, and a younger age of the modern Holarctic faunas.

## Introduction

The platygastroid wasps represent one of the underexplored territories of microhymenopteran diversity. With over 4000 described species and many more awaiting description, these frequently minute parasitoids may be found in virtually all habitats throughout the world and although many fine revisions have been forthcoming, the total number of species will still climb significantly in the coming years. In tandem with this systematic work, biological studies are needed, particularly as platygastroids are important egg and larva parasitoids for a wide diversity of insect and spider hosts, and may serve important roles in natural and agricultural ecosystems, acting as effective biological control agents in the latter. The lineage was particularly abundant during the Mesozoic, as they often represent the most numerous of hymenopteran inclusions in many of the world’s Cretaceous amber deposits (e.g., Grimaldi et al. 2002, Grimaldi and Engel 2005, Perrichot et al. 2010, McKellar and Engel 2012, Ortega-Blanco et al. 2014, and pers. obs.). They also occur in Tertiary ambers, but not with same diversity as has been observed in the Cretaceous ambers (see Johnson et al. 2008 for alternate interpretation). General accounts of Tertiary platygastroids have largely focused on the middle Eocene amber of the Baltic region (e.g., Brues 1940, Szabó and Oehlke 1986).

Formal New World Tertiary records of platygastroid wasps have been confined to the Early Miocene amber-bearing strata of Mexico (Masner 1969), although there are a variety of additional specimens from the ambers of Chiapas and the Dominican Republic requiring study (pers. obs.). Material from further south and well within the South American continent has not previously been available. It is therefore of interest to note the recent discovery of a well-preserved individual of a scelionine wasp in a middle Miocene amber from Peruvian Amazonia, along with other organic inclusions (Antoine et al. 2006, Petrulevičius et al. 2011). Herein we describe a new species of *Macroteleia* Westwood and make comparisons between this species and its modern congeners. *Macroteleia* contains some of the largest scelionids and is a diverse genus, with over 130 species encompassing a largely pantropical distribution, but with some species in more temperate habitats (Masner 1976, Chen et al. 2013). Where known, species are egg parasitoids of Tettigoniidae (Chen et al. 2013, and references therein). Only few fossil species of the genus have been previously documented. *Macroteleia renatae* Szabó & Oehlke, was described from a single female preserved in middle Eocene (Lutetian) Baltic amber, while a second species, *Macroteleia veterna* Cockerell, from the Eocene of the Isle of Wight (Cockerell 1921), was recently transferred in the genus *Calotelea* Westwood (Antropov et al. 2014: 341, pl. 1, fig. 5).

## Material and methods

The study is based upon a single specimen preserved in amber from the Pebas Formation (Mollusc Zone MZ7, late Middle Miocene, ~12 Ma; Wesselingh et al. 2006) that is exposed on the eastern bank of the Amazon River in the Tamshiyacu locality, 30 km upstream of Iquitos in northeastern Peru. The age and paleobiota of this amber deposit are preliminarily overviewed by Antoine et al. (2006). Only a small amount of amber has been recovered to date from this deposit, thus the documented diversity of the fossil arthropod fauna is not very high. This deposit is significant already, as Miocene fossil insects are otherwise virtually unknown in South America. The only insect inclusion comprehensively described so far from this amber is a psychodid fly (Petrulevičius et al. 2011).

**Figure 1.**
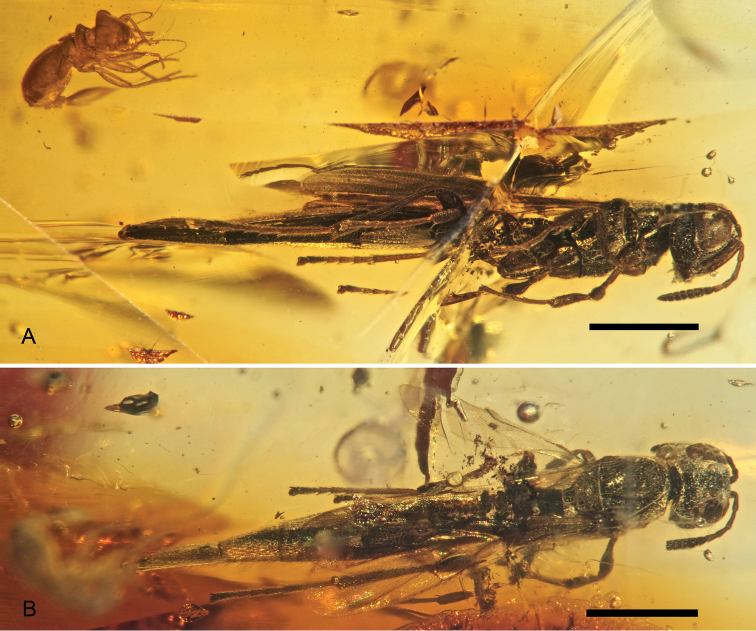
*Macroteleia yaguarum* Perrichot & Engel, sp. n., holotype MUSM-A-2006-4a, female **A** Right lateral habitus **B** Dorsal habitus. Scale bars: 1 mm.

The wasp specimen studied herein was originally preserved in a rather large (ca. 45 × 30 × 20 mm) piece of translucent yellow amber with seven syninclusions (two mites, one spider, one gall midge, two nymphal barklice, and the head of an ant). The piece was cut in eight smaller fragments each of which were polished to optimize the view of the different inclusions, and the scelionine wasp is now preserved in a small piece (12 × 5 × 3 mm) with one barklouse (Fig. 1A). Morphological terminology and the format for the description generally follow Masner (1976), Muesebeck (1977), Galloway (1978), Masner and Huggert (1989), Johnson and Masner (2006), Masner et al. (2007), Chen et al. (2013), and Ortega-Blanco et al. (2014). Photomicrographs were prepared using a Canon 5D Mark II digital camera attached to a Leica MZ APO stereomicroscope. Stacks of photographs taken at various depths of field were merged using HeliconFocus software (HeliconSoft Ltd.). Measurements were made using the ocular micrometer of the stereomicroscope.

## Systematic paleontology

### Genus *Macroteleia* Westwood, 1835

#### Macroteleia yaguarum

Taxon classificationAnimaliaHymenopteraPlatygastridae

Perrichot & Engel sp. n.

http://zoobank.org/8D3E135D-8273-4A15-A97D-BD4FDB0C76ED

Figs 1 –2

##### Type material.

Holotype MUSM-A-2006-4a, female, in amber fragment from the Pebas Formation (Mollusc Zone MZ7, late Middle Miocene, ~12 Ma; Wesselingh et al. 2006), Tamshiyacu locality, 30 km upstream of Iquitos, northeastern Peru (Antoine et al. 2006); deposited in the Paleontology Department of the Museo de Historia Natural, Universidad Nacional Mayor San Marcos, Lima, Peru (MUSM).

##### Diagnosis.

The new species can be characterized by the following combination of features: Antenna discolorous; flagellum with F1 elongate, nearly as long as F2+F3; clavus comprising six flagellomeres (F5–F10); face and vertex contiguously punctured; mesoscutum without median longitudinal carina, integument contiguously punctate; notauli not areolate or foveate; metapleuron and dorsal and ventral surfaces of metasoma largely rugulose punctate; metasoma elongate, integument largely rugulose punctate; tergum 4 (T4) laterally compressed and dorsally humped; integument largely dark brown to black, without areas of obviously yellowish or reddish maculation.

##### Description.

*Female*. Body length 5.23 mm; forewing length 2.70 mm, maximum width 0.73 mm; integument generally dark brown to black, wings subhyaline, veins dark brown; body elongate, cylindrical (Fig. 1A, B).

**Figure 2.**
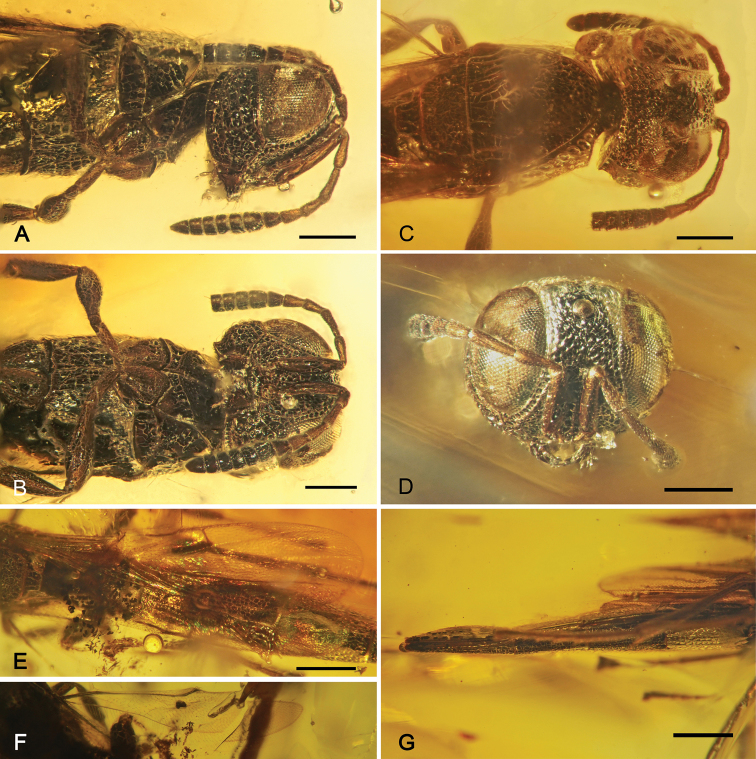
*Macroteleia yaguarum* Perrichot & Engel, sp. n., holotype MUSM-A-2006-4a, female **A** Head and mesosoma in lateral view **B** Head and mesosoma in ventral view **C** Head and mesosoma in dorsal view **D** Head in full face view **E** Forewing **F** Hind wing **G** Metasomal segments 4–6 in lateral view. Scale bars: **A–D:** 0.25 mm; **E–G:** 0.5 mm.

Head 0.55 mm long, 0.58 mm high, 0.74 mm wide, densely punctate, punctures large, appearing almost areolate, those of vertex contiguous, slightly smaller on face and gena (Fig. 2A–D); compound eyes oval, large, maximal diameter 0.43 mm, anteriorly bordered by a foveate groove; lateral ocelli well distant from each other, very close to inner margin of eyes; preoccipital ridge carinate, bordered anteriorly by foveate groove; antenna with 12 articles, discolorous, with scape, pedicel, and F1–F4 dark brown, remaining flagellomeres black; scape elongate, about 2.5× length of pedicel; pedicel longer than wide; F1 slightly longer than pedicel, three times as long as wide, finely microsetose; clavus comprising apical six flagellomeres (F5–F10), with basiconic sensilla on ventral surface distributed 2-2-2-2-2-1; mandibles small, each with three small teeth along apical margin.

Mesosoma 1.17 mm long, 0.65 mm wide. Pronotal dorsal surfaces lateral to mesoscutum with large areolate punctures arranged in two longitudinal rows, those closest to mesoscutum largest, anterior and lateral edges strongly carinate (Fig. 2C); pronotal lateral surface below carina impunctate, smooth. Mesoscutum with punctures similar to those of vertex, contiguous, without median longitudinal carina; notauli deeply impressed, not areolate, slightly wider posteriorly than anteriorly, converging posteriorly but not meeting, terminating at transscutal sulcus and well separated from each other; mesoscutellum sculptured as on mesoscutal disc, except posterior margin with single transverse row of large foveae. Netrion with anterior border composed of single dorsoventral row of posteriorly-opened areolae; mesopleuron with large, central, longitudinal depression, integument otherwise rugulose punctate, punctures nearly contiguous. Metanotum with single transverse row of large areolae, distinctly larger than those of posterior border of mesoscutellum; metapleuron with coarse, nearly contiguous rugulose punctures. Propodeum without armature, continuous medially. Legs imbricate and apparently impunctate; tibial spur formula 1-1-1; protibial calcar apically bifid; pretarsus with large arolium. Forewing membrane subhyaline (Fig. 2E); submarginal vein elongate, bearing a row of elongate setae; marginal vein elongate, as long as stigmal vein; postmarginal vein much longer than stigmal vein, total length greater than combined lengths of marginal and stigmal veins. Hind wing with complete vein bearing three distal hamuli apically, with well-defined posterior fringe of setae (Fig. 2F).

Metasoma elongate, 3.90 mm long, maximal width 0.50 mm, with narrow laterotergites; T2 about as long as T3; terga and sterna rugulose punctate, punctures arranged in loose rows, punctures separated by one puncture width, or more often less on S1–S3, punctures contiguous by S4 and onward; sterna without median longitudinal carina; T6 elongate, laterally compressed, dorsally humped, longitudinally striate (Fig. 2G).

*Male*. Unknown.

##### Etymology.

The specific epithet is a patronym for the native ethnic group Yagua, long-settled in the Tamshiyacu area, Maynas, Loreto.

##### Comments.

In Muesebeck’s (1977) key to New World species of *Macroteleia*, the fossil will run to *Macroteleia surfacei* Brues, a species from eastern North America. *Macroteleia yaguarum* sp. n. differs from *Macroteleia surfacei* most notably by the non-foveolate notauli (foveolate in *Macroteleia surfacei*), the contiguous punctures of the vertex (shagreened and impunctate on vertex in *Macroteleia surfacei*), and the continuous propodeum (medially divided propodeum in *Macroteleia surfacei*), among other details of integumental sculpturing (see Muesebeck 1977).

## Discussion

Although not the earliest fossil for this genus, the significance of the present individual rests in its demonstration of the occurrence of *Macroteleia* in the South American fauna during the Middle Miocene, i.e., well before a sustainable terrestrial contact with North America, and as the sole New World fossil of the clade. Unfortunately, it is impossible to accurately speculate on its possible relation to the diversity of living species given the absence of both a phylogenetic hypothesis for the genus, as well as the desperate need of a thorough modern revision of the South American fauna – a group of species which is certainly much more diverse than that documented by Muesebeck (1977). The occurrence of the genus in the early Neogene fauna of South America is not surprising given its considerable pantropical diversity, as well as its representation in the Eocene fauna of northern Europe. Pending a comprehensive phylogeny for the genus, biogeographic patterns remain speculative. Nonetheless, the very limited data tend to suggest a Cretaceous origin for the group, resulting in the presence of the genus in South America, tropical Africa, and Australia. In the early Paleogene, close contact between Africa and the southern archipelagos comprising Europe at the time would have permitted invasions into Europe, North America, and eastern Asia. The more ancient South American fauna would have then come into contact with those younger clades in North America during Great American Interchange around three million years ago. This hypothesis would mean that the South American, tropical African, and Australian faunas are relatively old within the genus, and that the modern Holarctic faunas are of younger origin, although subsequent migration of North American clades back into South America would be expected. It is hoped that renewed interest in platygastroid evolution and biology will provide eventually a comprehensive phylogeny for *Macroteleia*, at which time the known fossils may be integrated and the aforementioned gross patterns of biogeography that we suggest may be tested.

## Supplementary Material

XML Treatment for Macroteleia yaguarum
